# Association between discretionary screen time and depressive symptoms in Chinese adolescents: a cross-sectional study

**DOI:** 10.3389/fped.2026.1957282

**Published:** 2026-10-01

**Authors:** Zhi Xu, Shuye Yang, Shibin Wang, Xing Wang

**Affiliations:** 1 Department of Neurosis and Psychosomatic Diseases, The Third Hospital of Quzhou, Quzhou, Zhejiang, China; 2 Department of Neurology, Binzhou People’s Hospital, Binzhou, Shandong, China; 3 Guangdong Mental Health Center, Guangdong Provincial People’s Hospital (Guangdong Academy of Medical Sciences), Southern Medical University, Guangzhou, Guangdong, China; 4 Department of Neurosis and Psychosomatic Diseases, Huzhou Third Municipal Hospital, The Affiliated Hospital of Huzhou Normal University, Huzhou, Zhejiang, China

**Keywords:** adolescents, Chinese adolescents, cross-sectional study, depressive symptoms, discretionary screen time

## Abstract

**Background:**

Adolescents globally exhibit growing engagement with discretionary screen time, but its association with depressive symptoms remains unclear in developing economies.

**Objective:**

To examine the relationship between discretionary screen time and depressive symptoms among Chinese adolescents.

**Methods:**

A cross-sectional study of 4,706 adolescents in Maoming City, Guangdong Province, China. Participants were stratified into four discretionary screen time categories: <1 h (*n* = 1,372), 1–2 h (*n* = 1,277), 2–4 h (*n* = 1,032), and ≥4 h (*n* = 1,025). Depressive symptoms were assessed using the validated CESD-9, with a cutoff score of ≥10. The primary analysis was a multilevel logistic regression model with random intercepts for school and class, adjusted for all measured covariates except BMI, anxiety and insomnia; a model additionally adjusted for BMI, anxiety and insomnia served as a sensitivity analysis. Subgroup analyses evaluated potential effect modification.

**Results:**

The prevalence of depressive symptoms was 29.7% (1,398/4,706). In the primary multilevel model (random intercepts for school and class; adjusted for sociodemographic, socioeconomic and lifestyle factors, without BMI, anxiety and insomnia), compared with the <1 h reference group, the odds ratios were 1.21 (95% CI: 0.98–1.49; *p* = 0.082) for 1–2 h, 1.88 (95% CI: 1.52–2.33; *p* < 0.001) for 2–4 h, and 2.54 (95% CI: 2.04–3.16; *p* < 0.001) for ≥4 h; each screen-time category increment was associated with 39% higher odds of depressive symptoms (OR = 1.39, 95% CI: 1.29–1.49; *p* < 0.001). When additionally adjusted for BMI, anxiety and insomnia (sensitivity analysis, Model 4), the associations were attenuated but remained statistically significant for the higher categories (≥4 h: OR = 1.69, 95% CI: 1.28–2.22; per-category OR = 1.19, 95% CI: 1.09–1.29). Subgroup analyses revealed no statistically significant evidence of effect modification (all *p*-interaction >0.05).

**Conclusion:**

Prolonged discretionary screen time is significantly associated with higher odds of depressive symptoms among Chinese adolescents, indicating a graded association that strengthens with increasing screen time. These findings are relevant to public-health monitoring of discretionary screen use in adolescents.

## 1 Introduction

Depression represents a prevalent adolescent mental disorder globally, affecting 20%–30% of this population (1, 2). In China, youth mental health challenges have intensified amid mounting academic demands and ubiquitous digital immersion. Recent meta-analyses indicate a pooled depressive symptom prevalence of 26.2% among Chinese adolescents, with significantly elevated rates in secondary vs. primary students (3).

Concurrently, discretionary screen time has emerged as a critical public health concern among Chinese adolescents (4). The widespread availability of smartphones, tablets, and computers has dramatically transformed leisure activities, with adolescents increasingly engaging in screen-based entertainment such as watching movies and short videos, playing video games, and browsing social media platforms (e.g., WeChat Moments, Weibo) (5). National surveys indicate that Chinese adolescents spend substantial portions of their discretionary time on digital devices, with a considerable proportion exceeding recommended daily limits (6). Recent national surveys indicate that approximately 34.7%–38.9% of Chinese adolescents exceed the recommended screen time limits of no more than 2 h per day (7). A large-scale study of 27,006 students in Zhejiang Province found that 36.2% reported non-educational screen time of ≥2 h per day (8). Among Chinese children and adolescents aged 6–14 years, the average daily screen time was estimated at 2.77 h, with nearly half (46.4%) exceeding the 2 h threshold (9). These findings underscore the public health relevance of examining the association between discretionary screen time and mental health outcomes in this population. The World Health Organization and national guidelines have emphasized the importance of limiting discretionary screen time to promote adolescent well-being, given its potential negative associations with sleep quality physical activity, and psychosocial functioning (10, 11).

Accumulating evidence indicates robust associations between excessive discretionary screen exposure and higher odds of depressive symptoms in adolescents. Prolonged engagement with digital entertainment may be associated with depressive symptoms through multiple mechanisms: sleep cycle disruption (e.g., prolonged sleep latency, reduced duration), diminished in-person socialization, heightened cyberbullying/unrealistic social comparison exposure, and less time spent on physical activity and health-promoting behaviors (12, 13). Nevertheless, graded associations between specific discretionary screen time thresholds and depressive symptoms remain poorly quantified, particularly in Chinese youth cohorts. Methodologically, most extant studies conflate discretionary and academic screen use in aggregate metrics, potentially masking the distinct mental health implications of entertainment-focused digital consumption. Depression prevalence varies significantly across regions, with higher rates reported in rural and western regions compared to urban and eastern areas, highlighting the importance of examining screen time and depressive symptoms associations in diverse Chinese populations (14).

Consequently, this study delineates graded associations between discretionary screen time and depressive symptoms in Chinese adolescents. Leveraging a multi-district sample from Maoming City, we quantitatively assessed this relationship with comprehensive adjustment for key covariates.

## 2 Methods

### 2.1 Data source and study population

Data were obtained from the Adolescent Mental Health and Lifestyle Survey conducted in Maoming City, Guangdong Province, China (September 2024). Students were eligible if enrolled in primary or secondary school; the survey was implemented across grades 6–12 (primary grade 6 to senior high grade 3), and the final analytical sample comprised adolescents aged 8–20 years. To ensure that the sample was representative of the regional adolescent population, and taking into account the differences in economic development across regions as well as the feasibility of investigation, a multi-stage stratified systematic sampling method was adopted. The survey covered all administrative regions of Maoming City, including the municipal direct area (central urban districts), Maonan District, Dianbai District, Gaozhou City, Huazhou City, Xinyi City, and Binhai New Area. Sampling was proportionate to the number of enrolled students in primary schools, junior high schools, and senior high schools (or vocational/secondary technical schools) in each administrative region. Stratification was based on grade, covering seven grades from the sixth grade of primary school to the third year of senior high school (or the equivalent level), with approximately 1,724 students sampled per grade.

The sampling procedure was carried out in three stages. In the first stage, schools were selected as the primary sampling units: using a random-number table, 29 primary schools, 24 junior high schools, and 17 senior high/vocational schools were selected proportionally to the number of enrolled students in each region, yielding a total of 70 schools/school sections, which were designated as the survey sites of the Maoming Youth Mental Health Survey. In the second stage, classes were randomly drawn by grade within each sampled school according to the population size, with 65 classes selected from primary schools, 175 from junior high schools, and 163 from senior high schools, totaling 403 classes. In the third stage, approximately 28–32 students were selected within each sampled class using systematic sampling (e.g., the odd/even student-number method), yielding a total of 13,108 students who were sampled and invited to participate. (Supplementary Tables 3, 4).

Of the 13,108 students who were sampled and invited, 1,042 did not complete the interview or questionnaire and were excluded, leaving 12,066 students who completed the interview and questionnaire. Subsequent exclusion of 7,348 participants with missing CES-D or Discretionary Screen Time yielded 4,718 participants with complete CES-D and discretionary screen time data. After further excluding 12 individuals (0.25%) with incomplete covariate data (District, Gender, Age, Education, Residence, Birth order, Boarding status, Economy, Activity, Dietary, Myopia, BMI, Anxiety, Insomnia), the final analytical sample comprised 4,706 participants with complete data on all variables. (Figure 1).

**Figure 1:**
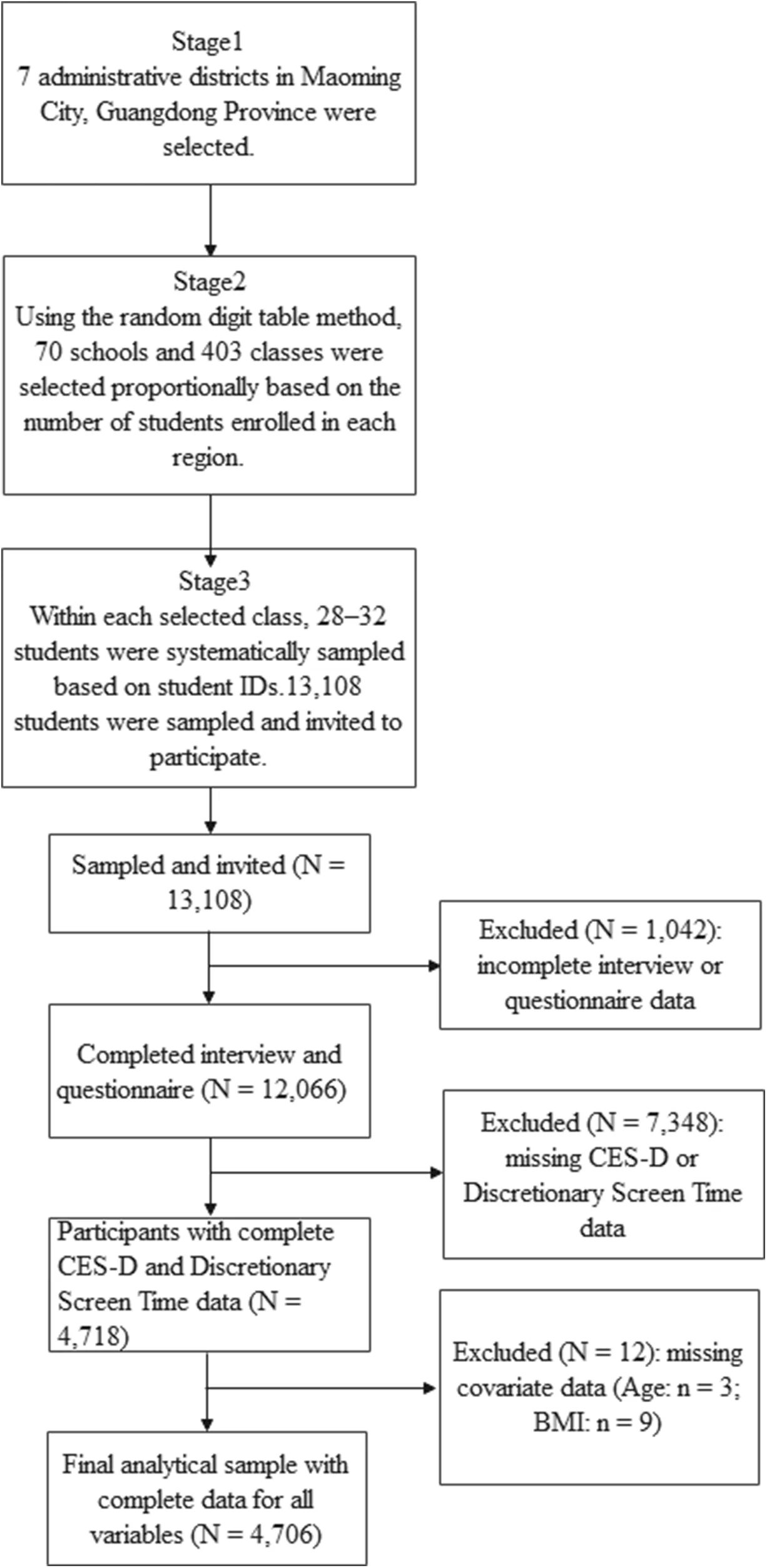
Study participant flowchart. Participant flow. Of 13,108 students sampled and invited through the three-stage sampling procedure, 12,066 completed the interview and questionnaire, 4,718 had complete CES-D and discretionary screen-time data, and 4,706 had complete data on all variables and were included in the analysis: 1,042 excluded: did not complete the interview or questionnaire. 7,348 excluded: missing CES-D or discretionary screen-time data. 12 excluded: missing covariate data (age, *n* = 3; BMI, *n* = 9). Final analytical sample: 4,706 participants.

### 2.2 Definition of depressive symptoms

Depressive symptoms refer to a constellation of emotional and somatic complaints, including depressed mood, anhedonia, diminished interest, fatigue, feelings of guilt or worthlessness, and disturbances in sleep and appetite. In epidemiological research, the 9-item Center for Epidemiologic Studies Depression Scale (CESD-9) can be used to assess depressive symptoms, with respondents reporting the frequency of these symptoms during the past week. Participants whose total CESD-9 score reached or exceeded the predefined cutoff were classified as having depressive symptoms. The CESD-9 is a validated screening instrument for depressive symptoms and does not constitute a clinical diagnosis of depressive disorder (15).

Depressive symptoms were measured using the Chinese adaptation of the Center for Epidemiological Studies Depression Scale-9 (CESD-9), psychometrically validated in national community (*n* = 30,801) and clinical (*n* = 415) cohorts. This instrument employs a 4-point Likert system (0 = rarely to 3 = most/all days) assessing symptom frequency over the preceding week. Total scores (range 0–27) were categorised descriptively as 0–9 (no or minimal symptoms), 10–16 (mild symptoms) and 17–27 (moderate-to-severe symptoms). For all analyses, the binary outcome “depressive symptoms” was defined as a CESD-9 total score ≥10, the validated screening cutoff (16). The CESD-9 is a screening instrument and does not constitute a clinical diagnosis of depressive disorder.

### 2.3 Definition of discretionary screen time

Discretionary Screen Time: Self-reported average daily hours spent using electronic screen devices (e.g., smartphones, computers, television) for entertainment purposes, including watching movies/TV shows/short videos, playing video games, and browsing social media platforms such as WeChat Moments and Weibo. Work- or study-related screen use was excluded.

Screen time was assessed using a single self-reported item asking about average daily time spent on electronic screen devices for recreational purposes, with categorical response options (<1 h, 1–2 h, 2–4 h, 4–6 h, 6–8 h, ≥8 h). Based on relevant literature, the ≥4 h/day categories were collapsed into a single group (8, 17).

### 2.4 Covariates

Covariates were selected based on documented or hypothesized links to discretionary screen time and depressive symptoms, serving as potential confounders. The covariates encompassed the following domains:

Demographics: District, gender, age, educational stage (categorized as: Grade 6 or below = primary school; Grades 7–9 = junior high school; Grades 10–12 = senior high school), residence (urban or rural), birth order (first-born, second-born, third-born, only child), and boarding status (day student or boarding student) (8).
Socioeconomic factors: Family economic status (self-reported as: very well-off, upper-middle, middle, lower-middle, difficult) (18).
Lifestyle behaviors:Physical activity frequency (every day or almost every day; 3–5 times/week; 1–2 times/week; 1–3 times/month; never/hardly ever). Dietary regularity (regular three meals per day; regular two meals per day; regular multiple meals per day [>3]; irregular) (19, 20).
Physical health indicators: Body mass index (BMI). Myopia severity (classified as: no myopia; mild myopia [spherical equivalent < −3.00 diopters (D), i.e., <300 degrees]; moderate to severe myopia [≥−3.00 D, i.e., ≥300 degrees]) (21).
Mental health: Anxiety symptoms and insomnia (22).

Anxiety symptoms were assessed using the Generalized Anxiety Disorder 7-item scale (GAD-7). Total GAD-7 scores range from 0 to 21, with higher scores indicating greater symptom severity (23). Insomnia severity was evaluated using the Insomnia Severity Index (ISI). Total ISI scores range from 0 to 28, with higher scores indicating greater severity (24).

Generalized variance inflation factors (GVIF) were calculated to assess multicollinearity among covariates (Supplementary Table 1). Although age and education showed relatively higher GVIF values (6.662 and 6.801, respectively), these were well below the conventional threshold of 10, indicating no severe multicollinearity. Both variables were retained in the final model due to their established theoretical importance as confounders in the screen time–depressive symptoms association.

E-values were calculated for the primary (Model 3) and sensitivity (Model 4) estimates, using school-level cluster-robust confidence limits. Because the outcome prevalence was 29.7%, the odds ratio overstates the risk ratio; following VanderWeele and Ding (25), the risk ratio was approximated as the square root of the OR before applying the E-value formula, and prevalence ratios were additionally estimated by modified Poisson regression with school-level robust standard errors. For the per-category association from Model 3 using school-level cluster-robust standard errors (OR: 1.37, 95% CI: 1.26–1.50), the E-value was 1.62 for the point estimate and 1.49 for the confidence limit; on the prevalence-ratio scale (PR: 1.23, 95% CI: 1.16–1.30), the corresponding E-values were 1.75 and 1.59 (Supplementary Table 9).

### 2.5 Statistical analysis

Because students were nested within classes and schools, we re-estimated the models using cluster-robust standard errors (clustered at the class and school levels) and computed class- and school-level intraclass correlation coefficients (ICCs) from an empty multilevel logistic model. In this study, “class proxy” is used as a proxy variable for the sampled classes. Due to the unavailability of actual class identifiers at the time of sampling, the management unit (the administrative class assigned by the school) was used as the class proxy for sampling and analysis purposes. Descriptive statistics characterized participant demographics. Categorical variables are expressed as frequencies (percentages), continuous variables as mean ± standard deviation or median (interquartile range). Group differences across Discretionary Screen Time categories were assessed using *χ*^2^ tests or ANOVA, and non-normally distributed continuous variables (such as GAD-7 and ISI) were compared using the Kruskal–Wallis test. Four multivariable logistic regression models evaluated the discretionary screen time-depressive symptoms association using the <1 h group (lowest exposure category) as the reference: Model 1: Unadjusted; Model 2: Adjusted for sociodemographic factors: district, gender, age; Model 3 (primary): Model 2 covariates plus education, residence, birth order, boarding status, family economic status, physical activity frequency (activity), dietary regularity (dietary), and myopia; Model 4 (sensitivity): Model 3 covariates plus BMI, anxiety (GAD-7) and insomnia (ISI). Because anxiety and insomnia may lie on the pathway between screen exposure and depressive symptoms, and BMI may likewise be affected by screen use, Model 3 is the primary model and Model 4 is presented as a sensitivity analysis. Because students were nested within class proxies and schools, the primary inference was based on a multilevel logistic regression model with random intercepts for school and class proxy, adjusted for the Model 3 covariates. The sequential Models 1–3 with model-based standard errors are shown in Table 1 to illustrate the pattern of attenuation. Model 4 (sensitivity analysis) is likewise presented with model-based standard errors in Table 2 to illustrate the pattern of attenuation. The primary model is additionally reported with school- and class-proxy-level cluster-robust standard errors (Supplementary Table 6). Subgroup analyses and E-value calculations used the Model 3 covariate set with school-level cluster-robust standard errors. Adjusted odds ratios (ORs) with 95% CIs were computed. All analyses used two-tailed tests with statistical significance at *p* < 0.05. Multilevel logistic models were fitted with lme4; cluster-robust variance estimates with sandwich and lmtest; prevalence ratios by modified Poisson regression with robust standard errors. Stratified analyses were conducted by gender, residence, body mass index, boarding status and insomnia. Effect estimates were visualized using forest plots, and *P* values for interaction were derived from likelihood ratio tests comparing models with and without cross-product terms. We follow VanderWeele and Ding (2017) in approximating the risk ratio as the square root of the odds ratio before applying the E-value formula (25). Statistical analyses were performed using R 4.2.2 (http://www.R-project.org, The R Foundation) and Free Statistics software version 2.1.1.

**Table 1:** Multivariable logistic regression analysis.

Variable		Model 1		Model 2		Model 3	
		OR (95%CI)	*P*-value	OR (95%CI)	*P*-value	OR(95%CI)	*P*-value
Screen time	4,706	1.68 (1.58–1.78)	<0.001	1.6 (1.5–1.71)	<0.001	1.37 (1.28–1.47)	<0.001
Screen time							
<1 h	1,372	1 (Ref)		1 (Ref)		1 (Ref)	
1–2 h	1,277	1.41 (1.17–1.71)	<0.001	1.3 (1.07–1.58)	0.009	1.22 (0.99–1.49)	0.058
2–4 h	1,032	2.73 (2.26–3.3)	<0.001	2.35 (1.93–2.86)	<0.001	1.9 (1.55–2.33)	<0.001
≥4 h	1,025	4.47 (3.71–5.39)	<0.001	3.87 (3.18–4.71)	<0.001	2.47 (1.99–3.05)	<0.001
Trend. test	4,706		<0.001		<0.001		<0.001

Model 1 was unadjusted.

Model 2 adjusted: Gender, Age, District.

Model 3 further adjusted for Model 2 covariates plus Education, Residence, Birth order, Boarding status, Economy, Physical activity frequency (Activity), Dietary regularity (Dietary), Myopia.

**Table 2:** Sensitivity analysis.

Variable		Model 4	
		OR (95%CI)	*P*-value
Discretionary Screen time	4,706	1.19 (1.09–1.29)	<0.001
Discretionary Screen time			
<1 h	1,372	1 (Ref)	
1–2 h	1,277	1.17 (0.9–1.52)	0.231
2–4 h	1,032	1.33 (1.02–1.73)	0.033
≥4 h	1,025	1.69 (1.28–2.22)	<0.001
Trend. test	4,706		<0.001

Model 4 Sensitivity analysis—adjusted: Gender, Age, District, Education, Residence, Birth order, Boarding status, Economy, Physical activity frequency (Activity), Dietary regularity (Dietary), Myopia, BMI, Anxiety, Insomnia.

## 3 Results

### 3.1 Characteristics of the participants

Of the 13,108 students who were sampled and invited, individuals with missing data on the exposure (discretionary screen time) or outcome (depressive symptoms) were excluded from the analysis. Among the covariates, missing values were minimal: age (*n* = 3, 0.06%) and BMI (*n* = 9, 0.19%). The exposure and outcome variables were the principal sources of missing data; with the exception of 12 participants, no covariate information was missing. Missingness of the exposure and outcome largely reflected item non-completion of the two key self-reported instruments — the 9-item CESD-9 and the single discretionary screen-time item — during a single-session, school-based questionnaire administration, and completion rates were markedly heterogeneous across survey sites (e.g., four schools with CESD-9 completion below 20%; Supplementary Table 5). These patterns indicate that administrative and logistical factors, rather than refusal driven by screen use or depressive symptoms, largely account for the missing exposure and outcome data.

Among the 8,402 excluded participants, we compared those included in the final analysis (*n* = 4,706) with those excluded (for whom basic demographic data were available for 7,360 participants) on key characteristics, including Gender, Age, District, Education, Residence, Birth order, Boarding status, Economy, Physical activity frequency (Activity), Dietary regularity (Dietary), Myopia, BMI, Anxiety, Insomnia. The results are summarized in Supplementary Table 7. However, all variables that differed between the two groups were measured at baseline and were included as covariates in our multivariable models. Among participants with complete exposure and outcome data, covariate missingness was minimal (<0.2% for all covariates, Supplementary Table 2); consequently, a complete-case analysis was performed. A total of 4,706 students were included, with discretionary screen time categorized into four groups: <1 h (*n* = 1,372), 1–2 h (*n* = 1,277), 2–4 h (*n* = 1,032), and ≥4 h (*n* = 1,025). We created the bar chart to more clearly illustrate the proportion in each group (Supplementary Figure 1). Approximately 43.7% of adolescents fell into the higher-usage groups (categories 2 and 3), while 56.3% were in the lower-usage groups (categories 0 and 1). The final analytical sample comprised participants aged 8–20 years. The vast majority of participants (99.94%) fell within the WHO-defined adolescent age range of 10–19 years, with only three participants outside this range (one aged 8 years; two aged 20 years). A sensitivity analysis excluding these three participants yielded substantially consistent results (Supplementary Table 8). Significant differences were observed across groups in most characteristics (all *p* < 0.05), except gender (*p* = 0.648) and birth order (*p* = 0.110).

Sociodemographic characteristics. The sample comprised 2,152 males (45.7%) and 2,554 females (54.3%). District distribution differed significantly (*p* < 0.001), with Maonan overrepresented in the <1 h group (20.8%) and underrepresented in the ≥4 h group (8.5%). Age and educational level showed marked gradients (both *p* < 0.001): younger students (≤13 years, 54.2%) and primary school students (27.3%) predominated in the <1 h group, whereas older students (16–17 years, 25.3% and 17.2%) and senior high school students (58.3%) were concentrated in the ≥4 h group. Rural residence was more common overall (56.1%) and slightly higher in the ≥4 h group (60.2%). Boarding status showed a strong inverse association (*p* < 0.001): day students comprised 42.1% of the <1 h group but only 18.7% of the ≥4 h group. Family economic status differed significantly (*p* = 0.011), though the majority across all groups reported middle-income status (57.4%).

Lifestyle and health-related characteristics. Physical activity frequency decreased with increasing screen time (*p* < 0.001): daily exercise fell from 31.9% (<1 h) to 12.1% (≥4 h), while sedentary behavior rose from 7.4% to 26.0%. Dietary regularity also deteriorated (*p* < 0.001): regular three meals decreased from 86.7% to 52.4%, and irregular eating increased from 3.6% to 20.9%. Myopia prevalence and severity increased significantly (*p* < 0.001). Mean BMI showed a modest increase (23.2 ± 8.5 vs. 24.3 ± 8.6, *p* = 0.004). Median anxiety scores (GAD-7) rose from 0.0 (IQR: 0.0–4.0) to 5.0 (IQR: 1.0–8.0), and insomnia severity (ISI) from 3.0 (IQR: 0.8–7.0) to 7.0 (IQR: 4.0–11.0) (both *p* < 0.001) (Table 3).

**Table 3:** Baseline characteristics.

Variables	Total (*n* = 4,706)	0 (*n* = 1,372)	1 (*n* = 1,277)	2 (*n* = 1,032)	3 (*n* = 1,025)	*p*
District, *n* (%)						<0.001
Binhai New Area	403 (8.6)	110 (8)	114 (8.9)	84 (8.1)	95 (9.3)	
Dianbai	1,308 (27.8)	350 (25.5)	319 (25)	303 (29.4)	336 (32.8)	
Gaozhou	693 (14.7)	187 (13.6)	203 (15.9)	148 (14.3)	155 (15.1)	
Huazhou	571 (12.1)	140 (10.2)	129 (10.1)	140 (13.6)	162 (15.8)	
Maonan	779 (16.6)	286 (20.8)	241 (18.9)	165 (16)	87 (8.5)	
Xinyi	952 (20.2)	299 (21.8)	271 (21.2)	192 (18.6)	190 (18.5)	
Gender, *n* (%)						0.648
Male	2,152 (45.7)	615 (44.8)	576 (45.1)	487 (47.2)	474 (46.2)	
Female	2,554 (54.3)	757 (55.2)	701 (54.9)	545 (52.8)	551 (53.8)	
Age (years), *n* (%)						<0.001
≤13	1,629 (34.6)	744 (54.2)	476 (37.3)	224 (21.7)	185 (18)	
14	684 (14.5)	189 (13.8)	189 (14.8)	156 (15.1)	150 (14.6)	
15	745 (15.8)	149 (10.9)	199 (15.6)	183 (17.7)	214 (20.9)	
16	737 (15.7)	104 (7.6)	158 (12.4)	216 (20.9)	259 (25.3)	
17	682 (14.5)	128 (9.3)	181 (14.2)	197 (19.1)	176 (17.2)	
≥18	229 (4.9)	58 (4.2)	74 (5.8)	56 (5.4)	41 (4)	
Education, *n* (%)						<0.001
Primary school	674 (14.3)	374 (27.3)	194 (15.2)	63 (6.1)	43 (4.2)	
Junior high school	2,036 (43.3)	656 (47.8)	589 (46.1)	407 (39.4)	384 (37.5)	
Senior high school	1,996 (42.4)	342 (24.9)	494 (38.7)	562 (54.5)	598 (58.3)	
Residence, *n* (%)						<0.001
Rural	2,641 (56.1)	709 (51.7)	736 (57.6)	579 (56.1)	617 (60.2)	
Urban	2,065 (43.9)	663 (48.3)	541 (42.4)	453 (43.9)	408 (39.8)	
Birth order, *n* (%)						0.11
First-born	1,886 (40.1)	565 (41.2)	499 (39.1)	407 (39.4)	415 (40.5)	
Second-born	1,647 (35.0)	504 (36.7)	447 (35)	337 (32.7)	359 (35)	
Third-born	947 (20.1)	240 (17.5)	263 (20.6)	239 (23.2)	205 (20)	
Only child	226 (4.8)	63 (4.6)	68 (5.3)	49 (4.7)	46 (4.5)	
Boarding status, *n* (%)						<0.001
Day student	1,485 (31.6)	577 (42.1)	428 (33.5)	288 (27.9)	192 (18.7)	
Boarding student	3,221 (68.4)	795 (57.9)	849 (66.5)	744 (72.1)	833 (81.3)	
Economy, *n* (%)						0.011
Very well-off	43 (0.9)	21 (1.5)	9 (0.7)	4 (0.4)	9 (0.9)	
Upper-middle	342 (7.3)	115 (8.4)	104 (8.1)	59 (5.7)	64 (6.2)	
Middle	2,701 (57.4)	797 (58.1)	729 (57.1)	594 (57.6)	581 (56.7)	
Lower-middle	1,187 (25.2)	309 (22.5)	317 (24.8)	282 (27.3)	279 (27.2)	
Difficult	433 (9.2)	130 (9.5)	118 (9.2)	93 (9)	92 (9)	
Activity, *n* (%)						<0.001
Every day or almost every day	970 (20.6)	437 (31.9)	269 (21.1)	140 (13.6)	124 (12.1)	
3–5 times/week	542 (11.5)	174 (12.7)	167 (13.1)	105 (10.2)	96 (9.4)	
1–2 times/week	1,677 (35.6)	472 (34.4)	519 (40.6)	409 (39.6)	277 (27)	
1–3 times/month	932 (19.8)	187 (13.6)	235 (18.4)	249 (24.1)	261 (25.5)	
Never/hardly ever exercise	585 (12.4)	102 (7.4)	87 (6.8)	129 (12.5)	267 (26)	
Dietary, *n* (%)						<0.001
Regular three meals per day	3,487 (74.1)	1,189 (86.7)	1,043 (81.7)	718 (69.6)	537 (52.4)	
Regular two meals per day	504 (10.7)	53 (3.9)	102 (8)	140 (13.6)	209 (20.4)	
Regular multiple meals per day (>3)	307 (6.5)	80 (5.8)	77 (6)	85 (8.2)	65 (6.3)	
Irregular	408 (8.7)	50 (3.6)	55 (4.3)	89 (8.6)	214 (20.9)	
Myopia, *n* (%)						<0.001
No myopia	1,702 (36.2)	623 (45.4)	450 (35.2)	329 (31.9)	300 (29.3)	
Myopia within 300 degrees	1,845 (39.2)	507 (37)	524 (41)	421 (40.8)	393 (38.3)	
Myopia 300 degrees or above	1,159 (24.6)	242 (17.6)	303 (23.7)	282 (27.3)	332 (32.4)	
BMI, Mean ± SD	23.7 ± 8.5	23.2 ± 8.5	23.4 ± 8.4	23.9 ± 8.5	24.3 ± 8.6	0.004
Anxiety (GAD-7), Median (IQR)	2.0 (0.0, 7.0)	0.0 (0.0, 4.0)	2.0 (0.0, 6.0)	4.0 (0.0, 7.0)	5.0 (1.0, 8.0)	<0.001
Insomnia (ISI), Median (IQR)	5.0 (2.0, 8.0)	3.0 (0.8, 7.0)	4.0 (1.0, 7.0)	6.0 (3.0, 9.0)	7.0 (4.0, 11.0)	<0.001

a Participants were ranked by discretionary screen time: 0 = <1 h, 1 = 1–2 h, 2 = 2–4 h, 3 = ≥4 h.

ISI, Insomnia severity index, range 0–28; GAD-7, generalized anxiety disorder 7-item scale, range 0–21.

### 3.2 Discretionary screen time is associated with depressive symptoms

Table 1 presents the results of the multivariable logistic regression analyses using standard (non-clustered) estimation; the multilevel model with random intercepts for school and class proxy is reported as the primary analysis below and in Supplementary Table 6. In the crude model (Model 1), each one-category increase in discretionary screen time was associated with a 68% higher odds of depressive symptoms (OR = 1.68, 95% CI: 1.58–1.78, *p* < 0.001). Compared with the <1 h reference group, the ORs were 1.41 (95% CI: 1.17–1.71) for 1–2 h, 2.73 (95% CI: 2.26–3.30) for 2–4 h, and 4.47 (95% CI: 3.71–5.39) for ≥4 h.

After adjustment for demographic variables (Model 2), the associations were modestly attenuated but remained highly significant: per-category OR = 1.60 (95% CI: 1.50–1.71, *p* < 0.001); categorical ORs: 1.30 (1.07–1.58), 2.35 (1.93–2.86), and 3.87 (3.18–4.71), respectively.

After further adjustment for the Model 2 covariates plus education, residence, birth order, boarding status, family economic status, physical activity frequency, dietary regularity, and myopia (Model 3), the associations were additionally attenuated. The per-category OR was 1.37 (95% CI: 1.28–1.47, *p* < 0.001), and the categorical ORs were 1.22 (95% CI: 0.99–1.49, *p* = 0.058), 1.90 (95% CI: 1.55–2.33), and 2.47 (95% CI: 1.99–3.05) for 1–2, 2–4, and ≥4 h, respectively.

In the fully adjusted model (Model 4), which additionally controlled for BMI, anxiety, and insomnia, the associations were further attenuated but remained statistically significant for the higher exposure categories: per-category OR = 1.19 (95% CI: 1.09–1.29, *p* < 0.001); categorical ORs: 1.17 (0.90–1.52, *p* = 0.231) for 1–2 h, 1.33 (1.02–1.73, *p* = 0.033) for 2–4 h, and 1.69 (1.28–2.22, *p* < 0.001) for ≥4 h. The linear trend was significant across all models (*p* < 0.001).

Intraclass correlation coefficients: Supplementary Table 5 reports latent-scale intraclass correlation coefficients from empty multilevel logistic models. The school-level ICC was 0.105 in a two-level model. In a three-level model the school-level ICC was 0.085 and the class-proxy ICC within schools was 0.068, so that two pupils in the same class proxy shared an ICC of 0.152. The prevalence of depressive symptoms (CESD-9 ≥10) ranged from 11.6% to 52.9% across the 30 schools contributing at least 50 analysed pupils. After adjustment for the Model 3 covariates the residual ICCs fell to 0.025 at the school level and 0.044 at the class-proxy level, indicating that much of the between-school variation is accounted for by the measured district, educational-stage and urban/rural composition of the schools.

Re-estimation allowing for clustering: Supplementary Table 6 shows the primary model (Model 3) estimated in four ways: as originally reported, with cluster-robust standard errors at the school level, with cluster-robust standard errors at the class-proxy level, and with a multilevel logistic model containing random intercepts for school and for class proxy. Clustering at the school level increased the standard errors of the exposure terms by 23% to 35%.

We now report the multilevel model as the primary clustering-aware analysis. It gave a per-category odds ratio of 1.39 (95% CI: 1.29 to 1.49); 1.21 (0.98 to 1.49) for 1–2 h vs. <1 h; 1.88 (1.52 to 2.33) for 2–4 h; and 2.54 (2.04 to 3.16) for ≥4 h.

### 3.3 Subgroup analyses: discretionary screen time and depressive symptoms

Subgroup analyses revealed positive associations between discretionary screen time and depressive symptoms across the examined strata (Figure 2). The adjusted ORs were 1.13 (95% CI: 1.00–1.29) for males and 1.28 (95% CI: 1.13–1.45) for females; 1.16 (1.03–1.31) for rural and 1.24 (1.09–1.41) for urban residents; 1.25 (1.20–1.29) for BMI <19.9 kg/m^2^ and 1.26 (1.22–1.30) for BMI ≥19.9 kg/m^2^; 1.32 (1.11–1.57) for day students and 1.16 (1.05–1.28) for boarding students; and 1.16 (1.03–1.30) for ISI <7 and 1.24 (1.09–1.42) for ISI ≥7. Collectively, all interaction *P* values exceeded 0.05, indicating no statistically significant evidence of effect modification by gender, residence, BMI status, boarding status, or insomnia severity. We note that non-significant interaction tests do not establish equivalence of effects across subgroups.

**Figure 2:**
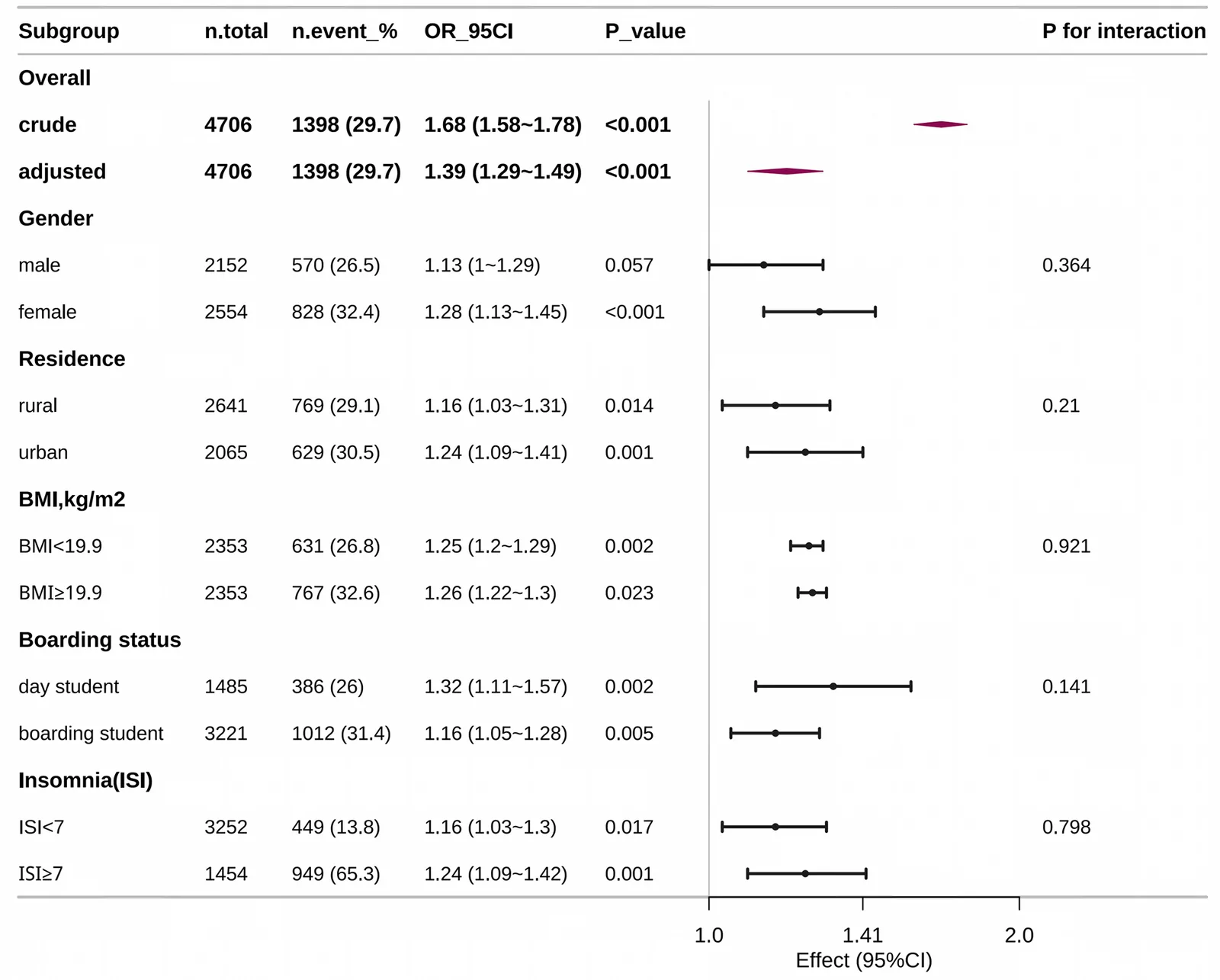
Subgroup analysis (forest plot). Subgroup analysis of the association between discretionary screen time and depressive symptoms. Odds ratios (ORs) per one-category increment and 95% confidence intervals (CIs) were estimated using multilevel logistic regression models adjusted for the Model 3 covariates; *P* values for interaction were derived from likelihood ratio tests. No statistically significant evidence of effect modification was observed across the examined subgroups.

## 4 Discussion

In this large cross-sectional study of 4,706 Chinese adolescents, we found a significant positive graded association between discretionary screen time and depressive symptoms. The association persisted after comprehensive adjustment for demographic, socioeconomic, and lifestyle covariates, with adolescents using screens for ≥4 h daily having 154% higher odds of depressive symptoms in the primary multilevel model (OR = 2.54, 95% CI: 2.04–3.16) compared with those using screens for <1 h. Subgroup analyses revealed no statistically significant evidence of effect modification across gender, residence, BMI, boarding status, and insomnia severity.

Our findings are consistent with previous research documenting positive associations between discretionary screen time and depressive symptoms in adolescent populations. A meta-analysis by Hoare and colleagues (2016) reported that sedentary behaviors, including discretionary screen time, were associated with increased odds of depressive symptoms in youth (26). Similarly, research by Twenge and Joiner (2018) found that heavy new media screen time was associated with more depressive symptoms and suicide-related outcomes (27). However, our study extends this literature in several important ways.

First, we observed a monotonic graded association between discretionary screen time and depressive symptoms. This pattern is consistent with a positive graded association; the cross-sectional design precludes causal interpretation. Notably, even moderate discretionary screen use (2–4 h/day) was associated with significantly higher odds of depressive symptoms in the primary multilevel model (OR = 1.88, 95% CI: 1.52–2.33) and in the sensitivity model (OR = 1.33), consistent with current guidelines recommending <2 h/day of discretionary screen time (11). Because the 1–2 h/day comparison was not statistically significant, our data do not provide evidence that below this threshold the odds of depressive symptoms begin to rise, and they do not justify recommending stricter screen-time limits (28–30).

Second, the robustness of the association between discretionary screen time and depressive symptoms after comprehensive multivariable adjustment is noteworthy. The crude association (OR = 1.68 per category) was substantially attenuated after adjusting for gender, age, and district (OR = 1.60); after further adjusting for education, residence, birth order, boarding status, economy, physical activity frequency (Activity), dietary regularity (Dietary), and myopia (OR = 1.39); and after additionally adjusting for BMI, anxiety, and insomnia (OR = 1.19), yet remained statistically significant (28, 31). This progressive attenuation suggests that confounding by these factors partially explains the crude association, but an association persists after adjustment for these factors. The attenuation following adjustment for anxiety and insomnia is particularly informative, as these conditions frequently co-occur with depressive symptoms and may share underlying mechanisms with excessive discretionary screen use (29, 30, 32).

However, the residual association suggests that the relationship between discretionary screen time and depressive symptoms is not entirely explained by anxiety and sleep disturbance. Previous studies have proposed several potential pathways, including social isolation, reduced physical activity, cyberbullying exposure, and neurobiological effects of prolonged digital stimulation (13, 33–37),though these mechanisms were not directly examined in the present study and warrant confirmation through longitudinal and experimental designs.

Third, we found no statistically significant evidence of effect modification across the examined subgroups. This contrasts with some previous studies that have suggested stronger associations in females (Kleidermacher et al., 2023) (38) or urban residents (Lin et al., 2022) (39). Our findings do not provide evidence of effect modification, which is consistent with the implementation of universal screen time recommendations rather than targeted interventions for specific demographic groups. However, non-significant interaction tests do not establish equivalence of effects across subgroups, and several subgroup-specific estimates were imprecise; accordingly, we do not claim strong generalizability across subgroups until confirmed in larger and more diverse populations.

Several mechanisms may explain why excessive discretionary screen time is linked to depressive symptoms. The time displacement hypothesis proposes that hours spent on screens may displace activities such as physical exercise, face-to-face socializing, and sleep (13). Our findings are compatible with this hypothesis, since the association weakened after adjusting for physical activity, although a cross-sectional design cannot test it directly. Social comparison may also play a role: adolescents exposed to idealized images on social media may develop negative self-perceptions and body dissatisfaction during this identity-forming period (40). In addition, the passive consumption of rapidly changing digital content may overload attention systems and reduce tolerance for delayed rewards, both important for emotional regulation. Blue light exposure from screens has been associated with melatonin suppression and circadian disruption, and sleep problems are in turn strongly associated with depressive symptoms (41). These pathways, proposed in the cited literature, were not examined in the present study.

### 4.1 Limitations and future directions

Our study has several strengths. The large, population-based sample from multiple districts enhances generalizability. The comprehensive assessment of potential confounders, including detailed socioeconomic, lifestyle, and mental health variables, minimizes residual confounding. The progressive adjustment strategy provides insight into the relative contributions of different confounder classes.

Several limitations should be acknowledged. The cross-sectional design precludes determination of temporal sequence; it is possible that adolescents with depressive symptoms are more likely to engage in excessive screen use as a coping mechanism or due to reduced motivation for alternative activities (reverse causation). Longitudinal studies are needed to clarify the direction of causality.

Screen time was assessed using a single self-reported item asking about average daily time spent on electronic screen devices for recreational purposes. The questionnaire asked participants to report their “average daily” screen time without specifying a particular recall period. While this approach may introduce some recall bias, it is consistent with methods used in previous large-scale Chinese adolescent studies (8, 19). Our instrument did not differentiate between weekday and weekend use, nor did it assess specific screen activity types (e.g., gaming, social media, video viewing) separately. However, similar single-item measures have been widely used in adolescent mental health research, and our findings are consistent with studies employing more detailed assessment tools (42, 43). The study was conducted in a single city in southern China, and generalizability to other regions or countries with different cultural and technological contexts may be limited. Additionally, due to the unavailability of the actual class identifiers at the time of sampling, the management unit served as a proxy in the sampling framework.

Missing data were present in both the exposure (discretionary screen time) and outcome (depressive symptoms) variables. The exclusion of participants with incomplete data may have introduced selection bias if missingness was associated with unmeasured factors related to both discretionary screen-time and mental health, potentially affecting the generalizability and validity of our observed associations.

Future research should: Conduct longitudinal cohort studies to establish the temporal sequence between screen time and depressive symptoms. Implement randomized controlled trials of screen time reduction interventions in school settings. Validate findings in diverse geographical and ethnic populations. Future research should utilize more detailed assessment instruments, including separate weekday/weekend measures and activity-specific screen time categories.

## 5 Conclusions

In conclusion, this large cross-sectional study found a significant, graded association between discretionary screen time and depressive symptoms (CESD-9 ≥10) among Chinese adolescents. The association persisted after adjustment for measured confounders and after accounting for clustering within schools and classes, and we found no statistically significant evidence of effect modification across demographic and clinical subgroups, although non-significant interaction tests do not establish equivalence across subgroups. These findings contribute to the growing evidence base on the association between screen time and adolescent mental health, supporting the public health relevance of monitoring discretionary screen use. Longitudinal and experimental studies are needed to establish causality and to evaluate whether reductions in discretionary screen time are associated with improvements in depressive symptoms.

## Data Availability

The raw data supporting the conclusions of this article will be made available by the authors, without undue reservation.

## References

[1] Racine N McArthur BA Cooke JE Eirich R Zhu J Madigan S. Global prevalence of depressive and anxiety symptoms in children and adolescents during COVID-19: a meta-analysis. JAMA Pediatr. (2021) 175(11):1142–50. 10.1001/jamapediatrics.2021.2482 34369987 PMC8353576

[2] GBD MDC. Global, regional, and national burden of 12 mental disorders in 204 countries and territories, 1990–2019: a systematic analysis for the global burden of disease study 2019. Lancet Psychiatry. (2022) 9(2):137–50. 10.1016/S2215-0366(21)00395-3 35026139 PMC8776563

[3] Zhou J Liu Y Ma J Feng Z Hu J Hu J. Prevalence of depressive symptoms among children and adolescents in China: a systematic review and meta-analysis. Child Adolesc Psychiatry Ment Health. (2024) 18(1):150. 10.1186/s13034-024-00841-w 39563377 PMC11577650

[4] Guo MM Koh KT Wang XZ. Trends of physical activity and recreational screen time among Chinese children and adolescents: a national study from 2017 to 2019. BMC Public Health. (2024) 24(1):1305. 10.1186/s12889-024-18822-1 38741155 PMC11092243

[5] Guo M Zhu Y Wang X. Physical activity and recreational screen time among Chinese children and adolescents: a national cross-sectional study. Front Public Health. (2024) 12:1376330. 10.3389/fpubh.2024.1376330 39050614 PMC11266031

[6] Lewis RYCH. Evidence Review of Screen Use in Childhood. Singapore: Saw Swee Hock School of Public Health, National University of Singapore (2023).

[7] Cai Y Zhu X Wu X. Overweight, obesity, and screen-time viewing among Chinese school-aged children: national prevalence estimates from the 2016 physical activity and fitness in China-the youth study. J Sport Health Sci. (2017) 6:404–9. 10.1016/j.jshs.2017.09.002 30356639 PMC6189269

[8] Wang H Bragg F Guan Y Zhong J Li N Pan J. Association between duration of electronic screen use for non-educational purposes and depression symptoms among middle and high school students: a cross-sectional study in Zhejiang Province, China. Front Public Health. (2023) 11:1138152. 10.3389/fpubh.2023.1138152 37261230 PMC10229063

[9] Qi J Yan Y Yin H. Screen time among school-aged children of aged 6–14: a systematic review. Glob Health Res Policy. (2023) 8(1):12. 10.1186/s41256-023-00297-z 37076910 PMC10113131

[10] Bull FC Al-Ansari SS Biddle S Borodulin K Buman MP Cardon G. World health organization 2020 guidelines on physical activity and sedentary behaviour. Br J Sports Med. (2020) 54(24):1451–62. 10.1136/bjsports-2020-102955 33239350 PMC7719906

[11] Friel CP Duran AT Shechter A Diaz KM. U.S. Children meeting physical activity, screen time, and sleep guidelines. Am J Prev Med. (2020) 59(4):513–21. 10.1016/j.amepre.2020.05.007 32863080 PMC7574791

[12] Hökby S Alvarsson J Westerlund J Carli V Hadlaczky G. Adolescents’ screen time displaces multiple sleep pathways and elevates depressive symptoms over twelve months. PLOS Global Public Health. (2025) 5(2):e0004262. 10.1371/journal.pgph.0004262 PMC11964217 40173157

[13] Guo H Man M Hu Z Liu Y Chen W Wang S. Screen time, physical activity, sleep, and depression risk in adolescents: an observational study based on the compositional isotemporal substitution model. Front Public Health. (2026) 14:1782512. 10.3389/fpubh.2026.1782512 42158198 PMC13182083

[14] Liu FR Song XQ Shang XP Wu MF Sui ML Dong YC. Meta-analysis of the detection rate of depressive symptoms among middle school students in China. Chin Ment Health J. (2020) 34(2):123–8.

[15] Lu LLT. Psychiatry. 9th ed Beijing: People’s Medical Publishing House (2024).

[16] He J Chen ZY Guo F Zhang J Yang YP Wang Q. Development of a short Chinese version of the center for epidemiologic studies depression scale. Chin J Behav Med Brain Sci. (2013) 22(12):1133–6. 10.3760/cma.j.issn.1674-6554.2013.12.023

[17] Imai S Close A Jones T Jones K. Excessive screen time among U.S. high school students: mental health, suicidal ideation and social image factors. Healthcare (Basel, Switzerland). (2025) 13:2833. 10.3390/healthcare13222833 41302221 PMC12652926

[18] Liao XT Huang JL Zou ZL Li ZY Li H. Association of family economic status and parent-child relationship with depressive symptoms in adolescents. Chin J Sch Health. (2025) 46(8):1111–5. 10.16835/j.cnki.1000-9817.2025255

[19] Lin Y Huang J-Y Rankin R McDonald S Jiang D-J Li S-X. Combined patterns of physical activity, screen-based sedentary behaviour, and sleep duration and their associations with depressive symptoms in Chinese adolescents. Front Public Health. (2025) 13:1691683. 10.3389/fpubh.2025.1691683 41341440 PMC12668928

[20] Yang RJ Lü SH Liu ZY Zhang X Liu ZH. Associations of screen time and dietary behaviors with depressive symptoms among junior high school students in five provinces of China. J Shanghai Jiaotong Univ Med Sci. (2024) 44(3):358–64. 10.3969/j.issn.1674-8115.2024.03.008

[21] Zong Z Zhang Y Qiao J Tian Y Xu S. The association between screen time exposure and myopia in children and adolescents: a meta-analysis. BMC Public Health. (2024) 24(1):1625. 10.1186/s12889-024-19113-5 38890613 PMC11186094

[22] Xu J Duan H Qin K Liu B. Association between screen time and depressive and anxiety symptoms among Chinese adolescents. Front Psychiatry. (2025) 16:1428885. 10.3389/fpsyt.2025.1428885 40395468 PMC12089052

[23] Spitzer RL Kroenke K Williams JBW Löwe B. A brief measure for assessing generalized anxiety disorder: the GAD-7. Arch Intern Med. (2006) 166:1092–7. 10.1001/archinte.166.10.1092 16717171

[24] Morin CM Belleville G Bélanger L Ivers H. The Insomnia Severity Index: psychometric indicators to detect insomnia cases and evaluate treatment response. Sleep. (2011) 34:601–8. 10.1093/sleep/34.5.601 21532953 PMC3079939

[25] VanderWeele TJ Ding P. Sensitivity analysis in observational research: introducing the E-value. Ann Intern Med. (2017) 167(4):268–74. 10.7326/M16-2607 28693043

[26] Hoare E Milton K Foster C Allender S. The associations between sedentary behaviour and mental health among adolescents: a systematic review. Int J Behav Nutr Phys Act. (2016) 13:108. 10.1186/s12966-016-0432-4 27717387 PMC5055671

[27] Twenge JM Joiner TE Rogers ML Martin GN. Increases in depressive symptoms, suicide-related outcomes, and suicide rates among U.S. Adolescents after 2010 and links to increased new media screen time. Clin Psychol Sci. (2018) 6(1):3–17. 10.1177/2167702617723376

[28] Deng Q Sha L Hou J Zhao X Xiang R Zhu J. Leisure screen time, internet gaming disorder, and mental health among Chinese adolescents: large-scale cross-sectional study. J Med Internet Res. (2026) 28:e80737. 10.2196/80737 41538793 PMC12856396

[29] Leung CY Torres R. Sleep duration does not mediate the association between screen time and adolescent depression and anxiety: findings from the 2018 National Survey of Children's Health. Sleep Med. (2021) 81:227–34. 10.1016/j.sleep.2021.02.031 33721600 PMC8499699

[30] Nagata JM Otmar CD Shim J Balasubramanian P Cheng CM Li EJ. Social media use and depressive symptoms during early adolescence. JAMA Netw Open. (2025) 8(5):e2511704. 10.1001/jamanetworkopen.2025.11704 40397441 PMC12096259

[31] Wang W Jiang J Qi L Zhao F Wu J Zhu X. Relationship between mental health, sleep status and screen time among university students during the COVID-19 pandemic: a cross-sectional study. BMJ Open. (2023) 13(8):e073347. 10.1136/bmjopen-2023-073347 38070905 PMC10729102

[32] Ahmed O Dawel A Walsh EI Burns RA Cherbuin N. Longitudinal associations between sleep and problematic social media use: mediating role of the motives for social media use. J Sleep Res. (2026) 35(1):e70254. 10.1111/jsr.70254 41309139

[33] Boers E Afzali MH Newton N Conrod P. Association of screen time and depression in adolescence. JAMA Pediatr. (2019) 173(9):853–9. 10.1001/jamapediatrics.2019.1759 31305878 PMC6632122

[34] Cao G Wei X Liu J Li X. The association between childhood trauma and adolescent cyberbullying: chain mediating roles of emotional intelligence and online social anxiety. Front Psychiatry. (2023) 14:1184382. 10.3389/fpsyt.2023.1184382 37324819 PMC10267877

[35] Liu M Wu L Yao S. Dose-response association of screen time-based sedentary behaviour in children and adolescents and depression: a meta-analysis of observational studies. Br J Sports Med. (2016) 50(20):1252–8. 10.1136/bjsports-2015-095084 26552416 PMC4977203

[36] Mohd Saat NZ Hanawi SA Hanafiah H Ahmad M Farah NMF Abdul Rahman NAA. Relationship of screen time with anxiety, depression, and sleep quality among adolescents: a cross-sectional study. Front Public Health. (2024) 12:1459952. 10.3389/fpubh.2024.1459952 39678241 PMC11638915

[37] Kazmi SM Jilani AQ Ahmad S Srivastava P Pandey K Anwar S. Effects of excessive social media use on neurotransmitter levels and mental health: a neurobiological meta-analysis. Era’s J Med Res. (2025) 12:56–60. 10.24041/ejmr.2025.11

[38] Kleidermacher LE Olfson M. Gender differences in the association between screen time and depression. AJPM Focus. (2023) 3:100176. 10.1016/j.focus.2023.100176 38304022 PMC10831171

[39] Lin Q Abbey C Zhang Y Wang G Lu J Dill S-E. Association between mental health and executive dysfunction and the moderating effect of urban-rural subpopulation in general adolescents from shangrao, China: a population-based cross-sectional study. BMJ Open. (2022) 12(1):e060270. 10.1136/bmjopen-2021-060270 35998954 PMC9403159

[40] Durkin SJ Paxton SJ. Predictors of vulnerability to reduced body image satisfaction and psychological wellbeing in response to exposure to idealized female media images in adolescent girls. J Psychosom Res. (2002) 53(5):995–1005. 10.1016/s0022-3999(02)00489-0 12445589

[41] Blazhkova A Czaja M Rehan D Rzym K Sitka H Solisch S. Blue light from screen and depression—a review. Zdrowie Publiczne. (2025) 135(1):49–56. 10.12923/2083-4829/2025-0010

[42] Wang Y Wu H Wu J Ma Y Wang J. Sleep quality moderated the mediating effect of BMI and waist circumference on the relationship between screen time and mental health in Chinese adolescents. Front Pediatr. (2025) 13:1602512. 10.3389/fped.2025.1602512 41050363 PMC12488706

[43] Matusova M Maracek M Pavelka J Ng K Medina C Tylsarova N. Measuring screen time among adolescents: test-retest reliability of HBSC questionnaire items across two countries. BMC Public Health. (2025) 26(1):290. 10.1186/s12889-025-25950-9 41413499 PMC12829222

