# Supplementary material for: Association between discretionary screen time and depressive symptoms in Chinese adolescents: a cross-sectional study
Source: Front Pediatr. 2026 Oct 1;14:1957282. doi: 10.3389/fped.2026.1957282 (PMC13669189; doi:10.3389/fped.2026.1957282)
Supplement: Supplementary file 1 [file Table1.docx]

****Supplementary Table 1. Covariate selection****

| **Term1** | **GVIF** |
| --- | --- |
| Crude | 1.208 |
| District | 1.686 |
| gender | 1.123 |
| Age | 6.662 |
| Education | 6.801 |
| Residence | 1.478 |
| Birth order | 1.096 |
| Boarding status | 1.643 |
| Economy | 1.152 |
| Activity | 1.199 |
| Dietary | 1.169 |
| Myopia | 1.154 |
| BMI | 1.019 |
| Anxiety | 1.114 |
| Insomnia | 1.102 |
