# Supplementary material for: Association between discretionary screen time and depressive symptoms in Chinese adolescents: a cross-sectional study
Source: Front Pediatr. 2026 Oct 1;14:1957282. doi: 10.3389/fped.2026.1957282 (PMC13669189; doi:10.3389/fped.2026.1957282)
Supplement: Supplementary file 2 [file Table2.docx]

****Supplementary Table 2. Missing data****

| **Variable** | **Miss.freq** | **Miss.percentage%** |
| --- | --- | --- |
| Activity | 0 | 0 |
| Age | 3 | 0.0636 |
| Anxiety | 0 | 0 |
| Birth order | 0 | 0 |
| BMI | 9 | 0.1908 |
| Dietary | 0 | 0 |
| District | 0 | 0 |
| Economy | 0 | 0 |
| Education | 0 | 0 |
| gender | 0 | 0 |
| Insomnia | 0 | 0 |
| Myopia | 0 | 0 |
| Residence | 0 | 0 |
| Boarding status | 0 | 0 |
