# Supplementary material for: Association between discretionary screen time and depressive symptoms in Chinese adolescents: a cross-sectional study
Source: Front Pediatr. 2026 Oct 1;14:1957282. doi: 10.3389/fped.2026.1957282 (PMC13669189; doi:10.3389/fped.2026.1957282)
Supplement: Supplementary file 3 [file Table3.docx]

**Supplementary Table 3. Monitoring Sampling of Adolescent Mental Health in Maoming City**

| **No.** | **District** | **School Name** | **School Type** |
| --- | --- | --- | --- |
| 1 | Maonan District (Municipal-direct) | Xiangyang Primary School | Primary School |
| 2 | Maonan District (Municipal-direct) | Weimin Road Primary School | Primary School |
| 3 | Maonan District (Municipal-direct) | No. 3 Primary School | Primary School |
| 4 | Maonan District (Municipal-direct) | Boya Middle School | Primary & Junior High School |
| 5 | Maonan District (Municipal-direct) | Longling School | Primary & Junior High School |
| 6 | Maonan District (Municipal-direct) | Tian Jiabing Middle School | Senior High School |
| 7 | Maonan District (Municipal-direct) | No. 16 Middle School | Junior & Senior High School |
| 8 | Maonan District | Shange Town Central Primary School | Primary School |
| 9 | Maonan District | Jintang Town No. 1 Middle School | Junior High School |
| 10 | Gaozhou City | Shigu Town Xiangshan Central School | Primary School |
| 11 | Gaozhou City | Tantou Town Tantou Central School | Primary School |
| 12 | Gaozhou City | Zhenjiang No. 1 Middle School | Junior High School |
| 13 | Gaozhou City | Shigu No. 1 Middle School | Junior High School |
| 14 | Gaozhou City | Nantang Middle School | Senior High School |
| 15 | Gaozhou City | Nanfang Vocational School | Vocational Senior High School |
| 16 | Huazhou City | Nansheng Sub-district Central Primary School | Primary School |
| 17 | Huazhou City | Boyang Town Central Primary School | Primary School |
| 18 | Huazhou City | Ligang Middle School | Junior High School |
| 19 | Huazhou City | Baoxu Middle School | Junior High School |
| 20 | Huazhou City | Hejiang Middle School | Senior High School |
| 21 | Huazhou City | Huazhou Vocational School | Vocational Senior High School |
| 22 | Xinyi City | Chidong Town Central Primary School | Primary School |
| 23 | Xinyi City | Huaixiang Central Primary School | Primary School |
| 24 | Xinyi City | Shuikou No. 1 Middle School | Junior High School |
| 25 | Xinyi City | Zhusha Middle School | Junior High School |
| 26 | Xinyi City | Zhenlong No. 1 Primary School | Primary School |
| 27 | Xinyi City | Beijie Town Xijiang Middle School | Junior High School |
| 28 | Xinyi City | Huaqiao Middle School | Junior & Senior High School |
| 29 | Xinyi City | Xinyi Vocational School | Vocational Senior High School |
| 30 | Dianbai District (Municipal-direct) | Maoming No. 1 Middle School | Junior & Senior High School |
| 31 | Dianbai District | Dianbai District No. 3 Middle School | Junior & Senior High School |
| 32 | Dianbai District | Xiadong Central Primary School | Primary School |
| 33 | Dianbai District | Dianbai No. 1 Primary School | Primary School |
| 34 | Dianbai District | Dianbai No. 7 Primary School | Primary School |
| 35 | Dianbai District | Dongcheng Middle School | Junior High School |
| 36 | Dianbai District | Chunhua School (Junior High Division) | Junior High School |
| 37 | Dianbai District | Dianhai Middle School | Senior High School |
