# Supplementary material for: Association between discretionary screen time and depressive symptoms in Chinese adolescents: a cross-sectional study
Source: Front Pediatr. 2026 Oct 1;14:1957282. doi: 10.3389/fped.2026.1957282 (PMC13669189; doi:10.3389/fped.2026.1957282)
Supplement: Supplementary file 4 [file Table4.docx]

**Supplementary Table 4. Sampling Framework of the Mental Health Monitoring of Adolescent Students in Maoming City**

| **District / County-level City** | **Code** | **Primary Schools* (No. of Classes)** | **Junior High Schools (No. of Classes)** | **Senior High / Vocational Schools (No. of Classes)** | **Total No. of Classes** | **Total No. of Students** |
| --- | --- | --- | --- | --- | --- | --- |
| The municipal direct area | A | 6 (14) | 5(35) | 4(38) | 87 | 2,830 |
| Maonan District | B | 6 (14) | 3 (21) | 2 (20) | 55 | 1789 |
| Gaozhou City | C | 2 (6) | 2 (16) | 2 (19)† | 41 | 1333 |
| Huazhou City | D | 5 (10) | 4 (30) | 2 (19)† | 59 | 1919 |
| Xinyi City | E | 5 (10) | 4 (29) | 2 (19)† | 58 | 1886 |
| Dianbai District | F | 3 (7) | 4 (30) | 4 (38) | 75 | 2439 |
| Binhai New Area | G | 2 (4) | 2 (14) | 1 (10) | 28 | 912 |
| **Total** |  | **29 (65)** | **24 (175)** | **17 (163)** | **403** | **13108** |

*Note: *The primary-school column header refers to primary-school sampling units; the table reports the planned number of schools and (in parentheses) the number of classes assigned at each school level. †In Gaozhou, Huazhou, and Xinyi, the senior high category may include vocational senior high schools. Districts with more favorable conditions may expand the sampling frame and sample size through the municipal mental health center.*
