# Supplementary material for: Association between discretionary screen time and depressive symptoms in Chinese adolescents: a cross-sectional study
Source: Front Pediatr. 2026 Oct 1;14:1957282. doi: 10.3389/fped.2026.1957282 (PMC13669189; doi:10.3389/fped.2026.1957282)
Supplement: Supplementary file 5 [file Table5.docx]

**Supplementary Table 5. Intraclass correlation coefficients for depressive symptoms (CESD-9 ≥10) (latent scale)**

| **Model** | **School-level ICC** | **Class-proxy ICC** | **Two pupils in the same class proxy** |
| --- | --- | --- | --- |
| Empty, two-level (pupils within schools) | 0.105 | — | 0.105 |
| Empty, three-level (pupils within class proxies within schools) | 0.085 | 0.068 | 0.152 |
| Adjusted for Model 3 covariates (residual) | 0.025 | 0.044 | 0.069 |
| Empty, three-level, excluding 4 schools with <20% CES-D completion (n = 4,514) | 0.072 | 0.068 | 0.141 |

ICC computed on the latent scale as τ² / (τ² + π²/3). Fitted with lme4 (Laplace approximation). The class proxy is the questionnaire-administration unit nested within school.
