# Supplementary material for: Association between discretionary screen time and depressive symptoms in Chinese adolescents: a cross-sectional study
Source: Front Pediatr. 2026 Oct 1;14:1957282. doi: 10.3389/fped.2026.1957282 (PMC13669189; doi:10.3389/fped.2026.1957282)
Supplement: Supplementary file 6 [file Table6.docx]

**Supplementary Table 6. Association between discretionary screen time and depressive symptoms, Model 3 (primary model), under four analytic approaches**

| **Approach** | **Per one-category increment** | **1–2 h vs <1 h** | **2–4 h vs <1 h** | **≥4 h vs <1 h** |
| --- | --- | --- | --- | --- |
| Ordinary logistic regression, model-based SE (as previously reported) | 1.37 (1.28–1.47) | 1.22 (0.99–1.49) | 1.90 (1.55–2.34) | 2.47 (2.00–3.05) |
| Cluster-robust SE, school level (42 clusters) | 1.37 (1.26–1.50) | 1.22 (1.01–1.46)† | 1.90 (1.44–2.50) | 2.47 (1.89–3.22) |
| Cluster-robust SE, class-proxy level (301 clusters) | 1.37 (1.28–1.48) | 1.22 (0.99–1.50) | 1.90 (1.52–2.37) | 2.47 (1.97–3.09) |
| Multilevel logistic regression, random intercepts for school and class proxy | 1.39 (1.29–1.49) | 1.21 (0.98–1.49) | 1.88 (1.52–2.33) | 2.54 (2.04–3.16) |
| Multilevel, excluding 4 schools with <20% CES-D completion (n = 4,514) | 1.39 (1.29–1.49) | 1.21 (0.98–1.50) | 1.88 (1.51–2.33) | 2.55 (2.03–3.19) |

Values are odds ratios (95% confidence intervals). All models adjusted for district, sex, age, educational stage, residence, birth order, boarding status, family economic status, physical activity, dietary regularity and myopia. † With 42 clusters the cluster-robust variance estimator is unstable for individual coefficients; the standard error of the 1–2 h term was smaller than its model-based counterpart, and we therefore draw no inference from that particular comparison under this approach.
