# Supplementary material for: Association between discretionary screen time and depressive symptoms in Chinese adolescents: a cross-sectional study
Source: Front Pediatr. 2026 Oct 1;14:1957282. doi: 10.3389/fped.2026.1957282 (PMC13669189; doi:10.3389/fped.2026.1957282)
Supplement: Supplementary file 7 [file Table7.docx]

**Supplementary Table 7 Sensitivity analysis (Included versus excluded participants)**

| **Variables** | **Total (n = 12066)** | **No Missing (n = 4706)** | **Missing (n = 7360)** | **p** |
| --- | --- | --- | --- | --- |
| **District, n (%)** |  |  |  | < 0.001 |
| Binhai New Area | 522 ( 4.3) | 403 (8.6) | 119 (1.6) |  |
| Dianbai | 1458 (12.1) | 1308 (27.8) | 150 (2) |  |
| Gaozhou | 787 ( 6.5) | 693 (14.7) | 94 (1.3) |  |
| Huazhou | 7135 (59.1) | 571 (12.1) | 6564 (89.2) |  |
| Maonan | 1039 ( 8.6) | 779 (16.6) | 260 (3.5) |  |
| Xinyi | 1125 ( 9.3) | 952 (20.2) | 173 (2.4) |  |
| **Gender, n (%)** |  |  |  | 0.001 |
| male | 5736 (47.5) | 2152 (45.7) | 3584 (48.7) |  |
| female | 6330 (52.5) | 2554 (54.3) | 3776 (51.3) |  |
| **Age（years）, n (%)** |  |  |  | < 0.001 |
| ≤13 | 2589 (21.5) | 1629 (34.6) | 960 (13.1) |  |
| 14 | 1836 (15.2) | 684 (14.5) | 1152 (15.7) |  |
| 15 | 2264 (18.8) | 745 (15.8) | 1519 (20.7) |  |
| 16 | 2303 (19.1) | 737 (15.7) | 1566 (21.3) |  |
| 17 | 2069 (17.2) | 682 (14.5) | 1387 (18.9) |  |
| ≥18 | 988 ( 8.2) | 229 (4.9) | 759 (10.3) |  |
| **Education, n (%)** |  |  |  | < 0.001 |
| primary school | 841 ( 7.0) | 674 (14.3) | 167 (2.3) |  |
| junior high school | 4805 (39.8) | 2036 (43.3) | 2769 (37.6) |  |
| senior high school | 6419 (53.2) | 1996 (42.4) | 4423 (60.1) |  |
| **Residence, n (%)** |  |  |  | 0.056 |
| rural | 6641 (55.0) | 2641 (56.1) | 4000 (54.3) |  |
| urban | 5425 (45.0) | 2065 (43.9) | 3360 (45.7) |  |
| **Birth order, n (%)** |  |  |  | < 0.001 |
| first-born | 4640 (38.5) | 1886 (40.1) | 2754 (37.4) |  |
| second-born | 4227 (35.0) | 1647 (35) | 2580 (35.1) |  |
| third-born | 2761 (22.9) | 947 (20.1) | 1814 (24.6) |  |
| only child | 438 ( 3.6) | 226 (4.8) | 212 (2.9) |  |
| **Boarding status, n (%)** |  |  |  | < 0.001 |
| day student | 4050 (33.6) | 1485 (31.6) | 2565 (34.9) |  |
| boarding student | 8016 (66.4) | 3221 (68.4) | 4795 (65.1) |  |
| **Economy, n (%)** |  |  |  | < 0.001 |
| very well-off | 72 ( 0.6) | 43 (0.9) | 29 (0.4) |  |
| upper-middle | 691 ( 5.7) | 342 (7.3) | 349 (4.7) |  |
| middle | 6806 (56.4) | 2701 (57.4) | 4105 (55.8) |  |
| lower-middle | 3324 (27.5) | 1187 (25.2) | 2137 (29) |  |
| difficult | 1173 ( 9.7) | 433 (9.2) | 740 (10.1) |  |
| **Activity, n (%)** |  |  |  | < 0.001 |
| Every day or almost every day | 1932 (16.0) | 970 (20.6) | 962 (13.1) |  |
| 3–5 times/week | 1279 (10.6) | 542 (11.5) | 737 (10) |  |
| 1–2 times/week | 4120 (34.1) | 1677 (35.6) | 2443 (33.2) |  |
| 1–3 times/month | 2639 (21.9) | 932 (19.8) | 1707 (23.2) |  |
| never/hardly ever exercise | 2096 (17.4) | 585 (12.4) | 1511 (20.5) |  |
| **Dietary, n (%)** |  |  |  | < 0.001 |
| regular three meals per day | 8498 (70.4) | 3487 (74.1) | 5011 (68.1) |  |
| regular two meals per day | 1461 (12.1) | 504 (10.7) | 957 (13) |  |
| regular multiple meals per day (>3) | 778 ( 6.4) | 307 (6.5) | 471 (6.4) |  |
| irregular | 1329 (11.0) | 408 (8.7) | 921 (12.5) |  |
| **Myopia, n (%)** |  |  |  | < 0.001 |
| no myopia | 3738 (31.0) | 1702 (36.2) | 2036 (27.7) |  |
| myopia within 300 degrees | 4730 (39.2) | 1845 (39.2) | 2885 (39.2) |  |
| myopia 300 degrees or above | 3598 (29.8) | 1159 (24.6) | 2439 (33.1) |  |
| **BMI, Mean ± SD** | 24.2 ± 8.6 | 23.7 ± 8.5 | 24.5 ± 8.7 | < 0.001 |
| **Anxiety(GAD-7), Median (IQR)** | 2.0 (0.0, 7.0) | 2.0 (0.0, 7.0) | 1.0 (0.0, 6.2) | 0.734 |
| **Insomnia(ISI), Median (IQR)** | 5.0 (2.0, 8.0) | 5.0 (2.0, 8.0) | 5.0 (2.0, 7.2) | 0.663 |

ISI = Insomnia Severity Index, range 0–28.

GAD-7 = Generalized Anxiety Disorder 7-item Scale, range 0–21.
