# Supplementary material for: Association between discretionary screen time and depressive symptoms in Chinese adolescents: a cross-sectional study
Source: Front Pediatr. 2026 Oct 1;14:1957282. doi: 10.3389/fped.2026.1957282 (PMC13669189; doi:10.3389/fped.2026.1957282)
Supplement: Supplementary file 8 [file Table8.docx]

**Supplement**ar**y Table **8 Sensitivity analysis excluding participants outside 10–19 years (n = 4,703)****

| **Variable** | | **OR (95%CI)** | **P value** |
| --- | --- | --- | --- |
| Discretionary Screen time | 4703 | 1.38 (1.28~1.47) | <0.001 |
| <1 h | 1372 | 1(Ref) |  |
| 1–2 h | 1276 | 1.21 (0.99~1.48) | 0.063 |
| 2–4 h | 1032 | 1.91 (1.55~2.34) | <0.001 |
| ≥4 h | 1023 | 2.48 (2~3.07) | <0.001 |
| Trend.test | 4703 |  | <0.001 |

Sensitivity analysis-Adjusted: Gender, Age, District, Education, Residence, Birth order, Boarding status, Economy, Physical activity frequency (Activity), Dietary regularity (Dietary), Myopia, BMI, Anxiety, Insomnia
