# Supplementary material for: Association between discretionary screen time and depressive symptoms in Chinese adolescents: a cross-sectional study
Source: Front Pediatr. 2026 Oct 1;14:1957282. doi: 10.3389/fped.2026.1957282 (PMC13669189; doi:10.3389/fped.2026.1957282)
Supplement: Supplementary file 9 [file Table9.docx]

**Supplementary Table 9. E-values for the association between discretionary screen time and depressive symptoms**

| **Estimate** | **OR (95% CI)†** | **E-value, point / CI limit** | **Adjusted PR (95% CI)†** | **E-value, point / CI limit** |
| --- | --- | --- | --- | --- |
| Model 3 (primary), per one-category increment | 1.37 (1.26–1.50) | 1.62 / 1.49 | 1.23 (1.16–1.30) | 1.75 / 1.59 |
| Model 3 (primary), 2–4 h vs <1 h | 1.90 (1.44–2.50) | 2.10 / 1.69 | 1.59 (1.32–1.92) | 2.56 / 1.97 |
| Model 3 (primary), ≥4 h vs <1 h | 2.47 (1.89–3.22) | 2.52 / 2.09 | 1.80 (1.51–2.14) | 2.99 / 2.39 |
| Model 4 (sensitivity), per one-category increment | 1.19 (1.08–1.31) | 1.40 / 1.23 | 1.12 (1.08–1.16) | 1.48 / 1.37 |

† Confidence intervals use school-level cluster-robust standard errors. For odds ratios the risk ratio was approximated as √OR before computing the E-value. Prevalence ratios were estimated by modified Poisson regression with school-level robust standard errors. The E-value for the confidence limit is 1 when the interval includes the null.
